# Ultrasound‐Microwave Associated Extraction of Anthocyanins From Mulberry and Their Antioxidant and Anticancer Activities

**DOI:** 10.1002/fsn3.71022

**Published:** 2025-10-03

**Authors:** Haibao Tang, Baogang Zhou, Ran Liu, Luo Weng, Kundian Che, Wei Gao, Zhanjun Chen, Jialin Yang, Haoyuan Luo, Shangjian Li, Wenzhong Hu

**Affiliations:** ^1^ School of Life Science Zhuhai College of Science and Technology Zhuhai, Guangzhou China; ^2^ School of Life Science Jilin University Changchun, Jilin China

**Keywords:** antioxidant, antitumor, mulberry anthocyanins, ultrasound‐microwave associated extraction

## Abstract

Mulberry anthocyanins (MA) are one of the important bioactive substances in mulberry, the extraction methods of MA are relatively limited, and there are few studies on their antitumor biological activity. This study employs response surface methodology (RSM) combined with single‐factor experiments to optimize the extraction conditions for ultrasound‐microwave associated extraction (UMAE) of MA. Based on the RSM, the optimal extraction process conditions for mulberry anthocyanins are as follows: solid–liquid ratio of 1:20, ethanol concentration of 80%, and ultrasonic power of 340 W. Under these conditions, the anthocyanin yield reached 5.98 mg/g. Biological activity, cell apoptosis, cell morphology, and cell invasion experiments all demonstrated that mulberry anthocyanins have an inhibitory effect on 4 T1 cells. These findings suggest that the extraction rate of anthocyanins from mulberry can be improved by UMAE, and it has also been demonstrated that mulberry anthocyanins possess notable antitumor activity, highlighting their potential for therapeutic applications. Antioxidant assays revealed that MA obtained via UMAE exhibited significantly stronger scavenging capacities against DPPH·, ·OH, and ABTS· radicals compared to conventional solvent extraction. This enhanced activity may be attributed to milder extraction conditions, which better preserve MA's structural integrity and bioactive properties. Theoretically, these results corroborate that MA possesses remarkable antioxidant potential, consistent with its previously reported antitumor efficacy.

## Graphical abstract

Ultrasound—microwave associated extraction (UMAE) optimized for mulberry anthocyanins (MA) gave a yield of 5.98 mg/g. MA showed strong antioxidant activity and inhibited 4 T1 cells, indicating potential therapeutic uses.

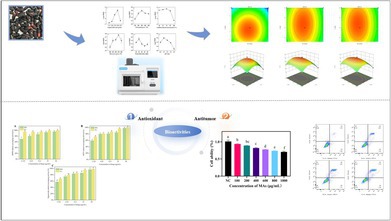

## 1 Introduction

Mulberry, a plant of the *Moraceae* family, is widely distributed in regions such as China, Japan, Africa, and North America (Bi et al. 2020). In China, it is classified as both a medicinal and food plant due to its diverse bioactive compounds, including vitamins, anthocyanins, flavonoids, and phenolic acids (Wen et al. 2019). Mulberry fruit offers numerous health benefits, such as antioxidant, anti‐cancer, anti‐inflammatory, hypoglycemic, liver‐protective, and neuroprotective properties (Lee and Kwon 2024; Zhou et al. 2024). While the anti‐tumor activities of anthocyanins from other berries, such as blueberries and wild cherries, are well documented, there is limited research on the anti‐breast cancer effects of mulberry anthocyanins.

Traditional methods of extracting mulberry anthocyanins, such as solvent extraction, are commonly used but are hindered by low efficiency, long extraction times, and high reagent consumption (Chen et al. 2018) (Xie et al. 2018). Moreover, the thermal instability of anthocyanins leads to reduced activity during these processes. To address these limitations, alternative methods have been developed, with optimization of extraction conditions being a critical step. Single‐factor experiments and response surface methodology (RSM) are widely used in optimizing extraction processes (Tang et al. 2025), as they systematically evaluate the effects of key parameters (e.g., solvent concentration, solid–liquid ratio, power, and time) on yield and bioactivity. For instance, Fan et al. (2019) applied single‐factor experiments combined with RSM to optimize polyphenol extraction from *Lonicera japonica* , demonstrating that this approach effectively identifies optimal conditions to maximize antioxidant activity. Similarly, Yoo et al. (2014) used RSM to optimize ajoene formation from garlic juice, highlighting the method's precision in modeling complex extraction systems. Zheng et al. (2019) further validated this by synchronously extracting polyphenols and polysaccharides from Tibetan tea, showing that RSM‐derived conditions significantly enhanced both yield and antioxidant capacity. Ultrasonic‐assisted extraction has been shown to improve efficiency (Al‐Baidhani et al. 2024; Zhang et al. 2019), while microwave‐assisted extraction achieves higher yields in a shorter time (Zannou et al. 2023). The combination of these techniques, known as UMAE, harnesses the cavitation effects of ultrasound and the thermal effects of microwaves to disrupt plant cell walls and enhance extraction efficiency (Tang et al. 2024). This method has the advantages of high efficiency, low energy consumption, maximizing the retention of the biological activity of MA, and short extraction time. Notably, advanced extraction techniques coupled with optimization methods have been successfully applied to various plant materials. Cinar Topcu et al. (2025) optimized ethanol extraction from sun‐dried apricots using artificial neural networks, a complement to RSM, and found that optimized extracts exhibited stronger antioxidant activity and inhibitory effects on HCT116 cells. Similarly, Wu et al. (2020) reported that optimized extraction of naphthoquinones from Onosma hookeri roots enhanced their antioxidant and anticancer activities, supporting the idea that extraction conditions directly influence the biological efficacy of plant‐derived compounds. In recent years, UMAE has been applied to the extraction of anthocyanins (Dong et al. 2022; Xue et al. 2021). However, no study has explored the use of UMAE for extracting MA with antioxidant and antitumor properties.

In this study, bioactive anthocyanins were extracted from mulberry using UMAE for the first time, followed by purification with AB‐8 macroporous resin. The antioxidant activity of the extracted anthocyanins was evaluated, alongside their effects on the viability, morphology, invasion, and apoptosis of 4 T1 breast cancer cells. Cho et al. (2017) investigated the mechanism of action of C3G, an anthocyanin found in mulberry, on MDA‐MB‐231 cells. They found that C3G can activate the Caspase‐3 enzyme and induce DNA fragmentation in live cells through the Bcl‐2 and Bax pathways, thereby initiating the apoptosis pathway and inhibiting the growth of cancer cells. Huang et al. (2011) found that mulberry extract, rich in anthocyanins, inhibits the growth of human gastric cancer cells (AGS) by inducing both intrinsic and extrinsic apoptosis through the activation of the p38/p53 and p38/c‐jun signaling pathways, demonstrating its crucial role in gastric cancer cell apoptosis. (Long et al. 2018) showed that MA significantly inhibits the progression of thyroid cancer by inducing apoptosis and autophagic cell death. The study analyzed the effects on SW1736 and HTh‐7 cells and found that MA inhibits the proliferation and migration of these cells. These findings provide new insights into the potential therapeutic applications of mulberry anthocyanins and demonstrate the efficacy of UMAE as an innovative extraction technique.

## 2 Materials and Methods

### 2.1 Materials and Reagents

Mulberry fruits were purchased from Guangdong Zhuhai Agricultural and By‐products Wholesale Logistics Center (Zhuhai, China) and stored at −20°C. Ethanol, hydrochloric acid, and ethyl acetate were purchased from Sinopharm Chemical Reagent Co. (Shanghai, China). Cornflower‐3‐O‐glucose and ascorbic acid were purchased from Shanghai Yuan Ye Co. Ltd. (Shanghai, China). DPPH·, ABTS·, and ·OH free radical kits were purchased from Nanjing Jian Co. Ltd. (Nanjing, China); CCK8 kit (Beyotime Biotechnology, China); Annexin V‐FITC/PI (Solarbio, Beijing, China); RPMI Medium 1640 basic (Gibco); fetal bovine serum (FBS); and 1% penicillin–streptomycin were supplied by other analytical grade chemical reagents were purchased from Xilong Chemical Co.

### 2.2 Extraction and Purification of MA

Mulberries were washed and then pulped in a blender. The pulp was subsequently freeze‐dried in a vacuum freezer for 72 h. After 500 g of mulberry fruits were pulped, they were placed in a beaker. The extraction was carried out using 1% HCl—70% ethanol (1:99, v/v) in the dark with an ultrasonic‐microwave associated extractor. This process adopted the synergistic extraction of ultrasound and microwave by using an ultrasonic‐microwave extraction instrument (XH‐300A). After extraction, the sample was centrifuged at 5000 rpm for 10 min (Fang et al. 2020). Then, the volume was fixed to the optimal volume with acidic ethanol to obtain the crude extract. The content of this extract was determined, and the optimal extraction method was obtained through the optimization of Response Surface Methodology (RSM). According to the optimized conditions, the concentrated solution of MA was obtained, and finally, it was adsorbed and purified by AB‐8 macroporous resin (Yan et al. 2016). After concentration and drying, MA was obtained.

### 2.3 One‐Factor Experiment for MA Extraction

#### 2.3.1 Liquid Solid Ratio Effect for Extraction

Five aliquots of mulberry samples were subjected to UMAE extraction under the following standardized conditions: simultaneous ultrasound‐microwave treatment (300 W each), acidified ethanol concentration (70%, v/v), extraction time (20 min), and pH (2.5). The liquid‐solid ratios were systematically varied (1:5, 1:10, 1:15, 1:20, and 1:25 g/mL) to evaluate extraction efficiency. All experiments were performed in triplicate with independent samples.

#### 2.3.2 Ultrasonic Power Effect for Extraction

Maintaining the core methodology from Section 2.3.1, we investigated ultrasonic power effects by testing five power levels (100, 200, 300, 400, and 500 W) while fixing the liquid‐to‐solid ratio at 1:15 g/mL. Other parameters remained unchanged (300 W microwave power, 70% acidified ethanol, 20 min extraction, pH 2.5).

#### 2.3.3 Effect of Acidified Ethanol Concentration

The extraction protocol was modified to examine solvent composition effects by testing ethanol concentrations (40%, 55%, 70%, 85%, and 100% v/v) acidified to pH 2.5. The optimal liquid‐solid ratio (1:15 g/mL) and ultrasonic power (300 W) determined in previous sections were employed, with other conditions matching Section 2.3.1.

#### 2.3.4 Microwave Power Optimization

Using the established optimal conditions (1:15 g/mL ratio, 300 W ultrasonic power, 70% acidified ethanol), we evaluated microwave power effects across five levels (200, 250, 300, 350, and 400 W). Extraction time (20 min) and pH (2.5) were maintained as in baseline protocols.

#### 2.3.5 Extraction Time Course Analysis

Building on results from previous optimizations, we fixed the microwave power at 350 W (determined optimal in 2.3.4) and tested extraction durations (20, 30, 40, 50, and 60 min) while maintaining other parameters (1:15 g/mL ratio, 300 W ultrasonic, 70% acidified ethanol, pH 2.5).

#### 2.3.6 pH‐Dependent Extraction Efficiency

The operation was the same as that in (2.3.4). Additionally, the microwave power for extraction was set at 350 W, the extraction time was set at 30 min, and the pH values were set at 0.5, 1.5, 2.5, 3.5, and 4.5, respectively.

### 2.4 Optimization of Anthocyanin Extraction

Based on the results of one‐way experiments, Box‐Behnken design was employed to explore the relationship between extraction conditions and anthocyanin yield (X), aiming to identify the optimal combination of variables. Design‐Expert software (version 13) was used for the fitting analysis. In this study, finally, the three factors A (ethanol concentration), B (ultrasonic power), and C (material‐liquid ratio) were used as the independent variables, and the extraction rate was used as the response indicator to perform a 3‐factor, 3‐level response surface experimental design (Table 1).

**TABLE 1:** Factors and levels of Box–Behnken design.

Factor	Level		
	−1	0	1
A Liquid–solid ratio/(g/mL)	15	20	25
B ethanol concentration/(%)	55	70	85
C Ultrasonic power/(W)	200	300	400

### 2.5 Determination of Total Anthocyanin Content

Total anthocyanin was determined by the pH parallax method. Absorbance values were detected at 520 and 700 nm, respectively. The total anthocyanin content was represented by the Equation (1) (Yan and Zheng 2017):

(1) $$\text{anthocyanin content}=\left[\frac{A\times \mathrm{MW}\times \mathrm{DF}\times V}{\beta \times W\times L}\right]$$

where *A* is the difference in absorbance, MW is the molecular weight of C3G, DF is the dilution factor, *V* is the volume, *T* is the molar extinction coefficient of C3G, *W* is the weight of mulberry, and *L* is the optical range.

### 2.6 Determination of Antioxidant Capacity In Vitro

#### 2.6.1 Measurement of DPPH· Clearance Capacity

Mulberry anthocyanins were prepared in concentrations of 3.125, 6.25, 12.5, 25, and 50 μg/mL. For the assay, 2.0 mL of anthocyanin and vitamin C solutions at various concentrations were mixed with 2.0 mL of DPPH solution (Kim et al. 2020). The absorbance at 517 nm was measured after 30 min of reaction; DPPH· clearance was calculated using the Equation (2):

(2) $$\text{DPPH}\cdot \left(\%\right)=\left[1-\frac{\left(A_{1}-A_{2}\right)}{A_{0}}\right]\times 100\%$$

where *A*_1_ is the absorbance value of the sample solution at different concentrations; *A*_2_ is the absorbance value of the sample itself (excluding DPPH·); *A*_0_ is the absorbance value of the DPPH· solution.

#### 2.6.2 Measurement of ABTS·· Free Radical Scavenging Rate

The appropriate ABTS· solution was mixed with potassium sulfate and placed at 4°C for 12 h. 3.125, 6.25, 12.5, 25, and 50 μg/mL of each of the mulberry anthocyanin and Vc solutions were taken for 0.1 mL, followed by the addition of 1.9 mL of the ABTS· radical solution, and then the absorbance at 734 nm was measured after 15 min of resting away from light (Liu et al. 2019). The ABTS·· clearance was calculated according to Equation (3):

(3) $$\text{ABTS}\cdot \left(\%\right)=\left[1-\frac{\left(A_{1}-A_{2}\right)}{A_{0}}\right]\times 100\%$$

where *A*_1_ is the absorbance value of the sample solution at different concentrations; *A*_2_ is the absorbance value of the sample itself; and *A*_0_ is the absorbance of anhydrous ethanol instead of the sample at 734 nm.

#### 2.6.3 Measurement of Hydroxyl Radical Scavenging Capacity

The MA solutions at concentrations of 3.125, 6.25, 12.5, 25, and 50 μg/mL were measured at 526 nm following the method described by Chen et al. (2019) for the hydroxyl radical scavenging capacity (·OH), with Vc as a reference. ·OH (%) was calculated using the Equation (4):

(4) $$\cdot \mathrm{OH}\;\left(\%\right)=\left[1-\frac{\left(A_{1}-A_{2}\right)}{A_{0}}\right]\times 100\%$$

where *A*_1_ is the absorbance value of the sample; *A*_2_ is the absorbance value of deionized water; *A*_0_ is the absorbance value of deionized water.

### 2.7 Studies on the Activity of MA on 4 T1 Cells

#### 2.7.1 Cell Culture

The 4 T1 cells were cultured at 37°C in a 90% RMPI 1640 medium supplemented with 9% fetal bovine serum (FBS) and 1% penicillin–streptomycin in an incubator with 5% CO_2_ (Ye et al. 2024).

#### 2.7.2 Cell Viability Assay

Cell viability was primarily assessed using the CCK8 assay. The experiment was divided into the treatment group, control group, and blank control group. Cells were seeded at a density of 1 × 10^5^ cells/mL in 96‐well plates. Different concentrations (0.1, 0.2, 0.4, 0.6, 0.8, 1, and 2 mg/mL) of MA were used to treat the control group, followed by incubation for 24 h (Stanca et al. 2024). Each experiment was performed in triplicate, and the cell inhibition rate (X%) was calculated using the following formula (Equation 5):

(5) $$X\%=\left(1-\frac{{\mathrm{OD}}_{1}}{{\mathrm{OD}}_{2}}\right)\times 100\%$$

where OD_1_ is the absorbance value of the experimentally administered group at absorbance 450 nm; OD_2_ is the absorbance value of the blank control group at absorbance 450 nm.

#### 2.7.3 Cytomorphological Observations

Logarithmically grown 4 T1 cells were taken and inoculated in 24‐well plates (2 × 10^5^/well). 500 μL of 0.25, 0.5, and 1 mg/mL of MA was added to the administered group, and the control group was cultured normally. The cell morphology was photographed by an inverted optical microscope 24 h later (Zhou et al. 2014).

#### 2.7.4 Wound Healing Experiment

4 T1 cells at a density of 1 × 10^6^ cells/mL were seeded into 6‐well plates, and uniform lines were marked on the bottom of the plate. After 24 h of culture, different concentrations of MA (0.25, 0.5, and 1 mg/mL) were added (Wu et al. 2018). After another 24 h of incubation, the extent of cell migration was observed using an inverted fluorescence microscope. Cell migration rate (C%) was calculated using ImageJ software, and the formula is as follows (Equation 6):

(6) $$C\%=\left(W_{0}-W_{1}\right)/W_{0}$$

where C % is cell mobility, *W*_0_ is initial scratch width, *W*_1_ is scratch width after culture.

#### 2.7.5 Apoptosis Assays

4 T1 cells were seeded in 6‐well plates at 4 × 10^5^/mL. The apoptosis was detected by the Annexin V‐FITC/PI apoptosis kit. Subsequently, the effect of 0.25, 0.5, and 1 mg/mL administration of MA on apoptosis was determined by flow cytometry, and the results were processed using Flow Jo (version 10.8.1).

### 2.8 Data Processing and Statistical Analysis

All results were presented as mean ± standard deviation, and all samples underwent at least 3 replicate experiments. Significant differences between the treatment and control groups were performed using SPSS software (version 17.0) by one‐way ANOVA and a mean difference significance *t*‐test. (*p* < 0.05) was considered significant for all tests compared to the blank or model groups.

## 3 Results and Discussion

### 3.1 Single Factor Experiment of Mulberry Anthocyanins

**FIGURE 1:**
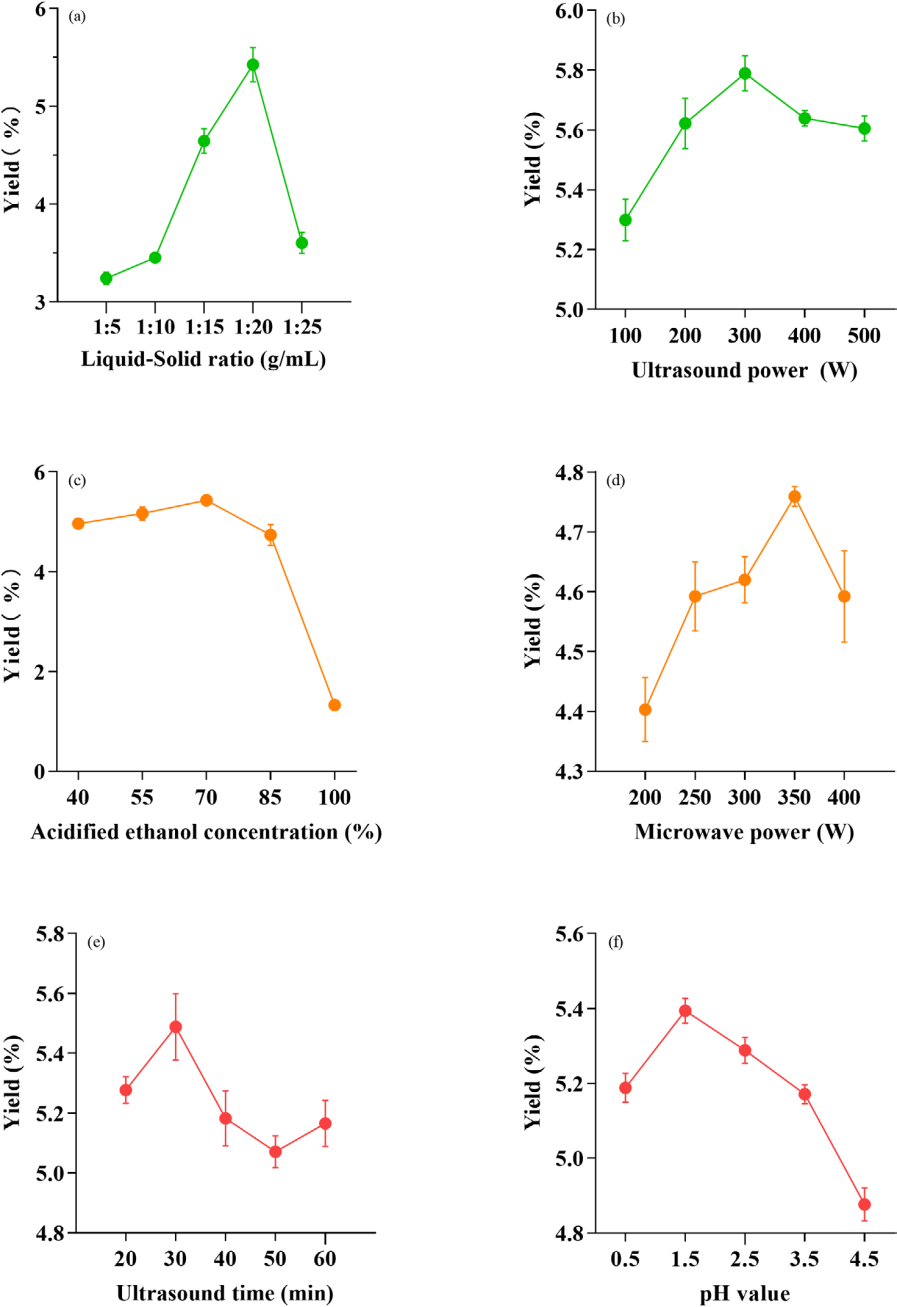
Effects of extraction parameters on MA yield: (a) Liquid–solid ratio (g/mL), (b) Ultrasonic power (W), (c) Ethanol concentration (%, v/v), (d) Microwave power (W), (e) Extraction time (min), (f) pH value, (*n* = 3). Value Error bars represent SD (*n* = 3).

Figure 1a–f present the results of a preliminary single‐factor study on the optimization of mulberry anthocyanins (MA) extraction rate, examining various parameters such as solid–liquid ratios, extraction time, ultrasonic power, microwave power, acidified ethanol concentration, and pH value. As depicted in Figure 1a, the optimal liquid–solid ratio for anthocyanin extraction is 1:20, beyond which the extraction rate declines. This suggests that while an adequate amount of solvent enhances anthocyanin solubility, an excess can lead to saturation, diminishing the extraction efficacy (Fang et al. 2020). Figure 1b indicates that the maximum anthocyanin extraction rate of 5.6 mg/g is achieved at an ultrasound power of 400 W, with a slight decrease observed at 500 W. This could be attributed to the increased local temperature at higher powers, accelerating anthocyanin degradation 21 (Trasanidou et al. 2016). As shown in Figure 1c, ethanol concentrations ranging from 40% to 100% were evaluated for their impact on anthocyanin extraction. Figure 1c shows that a 70% ethanol concentration yields the highest extraction rate of approximately 5.2 mg/g; concentrations above 70% reduce the solvent's polarity, decreasing anthocyanin solubility and thus the extraction rate (Panic et al. 2019). Figure 1d demonstrates that the extraction yield is highest at 4.7 mg/g with a microwave power of 350 W, with a decrease in yield at higher powers (Simić et al. 2016).

Moderate microwave power facilitates cell wall rupture, while excessive power can rapidly increase temperature, promoting the thermal degradation of anthocyanins. Regarding ultrasound time, as shown in Figure 1e, the extraction rate reaches a peak of 5.5 mg/g at 20 min of ultrasound treatment. Extended ultrasound durations result in a decrease in extraction volume, likely due to the initial effectiveness of ultrasound in breaking down cell walls and the subsequent rise in solution temperature, which can degrade anthocyanins (Chemat et al. 2012).

Figure 1f illustrates that the anthocyanin extraction yield reaches its maximum of 5.3 mg/g at pH 2.5, after which it declines significantly with an increase in pH. This phenomenon can be attributed to the increased stability of anthocyanins under acidic conditions and their susceptibility to degradation under alkaline conditions, leading to a loss in color and extraction efficiency (Barros and Ferreira 2017).

### 3.2 Analysis of Variance for Fitting the Model

Utilizing the Box–Behnken Design methodology, an experimental matrix (Table 2) was constructed, incorporating variables A, B, and C as influencing factors, with anthocyanin concentration serving as the measured output. Subsequently, a quadratic polynomial regression model was derived through computational analysis.

**TABLE 2:** Extraction results from different optimization groups.

No.	Factor			Yield (%)
	A/(g/mL)	B/(%)	C/(W)	
1	20	70	200	4.67
2	25	55	300	4.46
3	15	85	300	4.41
4	25	85	300	4.13
5	20	85	400	4.94
6	20	70	300	6.12
7	20	55	400	5.19
8	25	70	400	4.89
9	20	70	300	5.95
10	20	55	200	4.91
11	15	70	400	5.26
12	20	70	300	5.85
13	20	85	200	4.67
14	15	55	300	4.85
15	15	70	200	4.94
16	20	70	300	6.15
17	20	70	300	5.83

According to the data obtained from the 17 sets of experiments in the BBD optimization group above, a method analysis and a fitting analysis of the quadratic polynomial regression equation were carried out (Wang et al., n.d.). The detailed data are shown in Table 3. Finally, through calculation, the regression equation for the yield (%) of mulberry anthocyanins was obtained as follows:

$$\begin{array}{c} \text{Yield}\;\left(\%\right)=5.98-0.1637A-0.1575B+0.1363C+0.0275\mathrm{AB}\\ -0.0250\mathrm{AC}-0.0025\mathrm{BC}–0.7525A^{2}-0.7650B^{2}-0.2875C^{2} \end{array}$$

**TABLE 3:** ANOVA for quadratic regression models.

Source of variation	Square sum	Degrees of freedom	Mean square	*F*‐value	*p*	Significance
Modle	6.27	9	0.6967	49.40	< 0.0001	**
A	0.2145	1	0.2145	15.21	0.0059	**
B	0.1984	1	0.1984	14.07	0.0072	**
C	0.1485	1	0.1485	10.53	0.0142	*
AB	0.0030	1	0.0030	0.2145	0.6573	ns
AC	0.0025	1	0.0025	0.1773	0.6864	ns
BC	0.0000	1	0.0000	0.0018	0.9676	ns
A^2^	2.38	1	2.38	169.05	< 0.0001	**
B^2^	2.46	1	2.46	174.71	< 0.0001	**
C^2^	0.3480	1	0.3480	24.68	0.0016	**
Residual	0.0987	7	0.0141			
Lost proposal	0.0099	3	0.0033	0.1490	0.9251	ns
Pure error	0.0888	4	0.0222			
Total error	6.37	16				
	*R* ^2^ = 0.9845		*R*_adj_^2^ = 0.9646		C.V.% = 2.31	

*Note:* “*” is significant difference, *p* < 0.05; “**” is highly significant difference, *p* < 0.01; “ns” is not significant difference.

In order to verify the effectiveness of the model, the established mathematical model and its regression coefficient were analyzed for variance, and the results are shown in Table 2. It is not difficult to see from the table that the regression model reached a very significant level (*p* < 0.0001), and the misfit term was not significant (*p* > 0.05), and the fit was good (*R* ^2^ = 0.9845). The fitting equation also reflects the relationship between the ratio of solid–liquid, ethanol concentration and ultrasonic frequency on the extraction rate. In addition, A, B, and C in the primary term and A^2^, B^2^, and C^2^ in the quadratic items were extremely significant (*p* < 0.01). In conclusion, this model can be used for the analysis and prediction of the yield of anthocyanins extracted from mulberry by ultrasonic‐microwave method.

### 3.3 Response Surface Optimization and Model Validation

**FIGURE 2:**
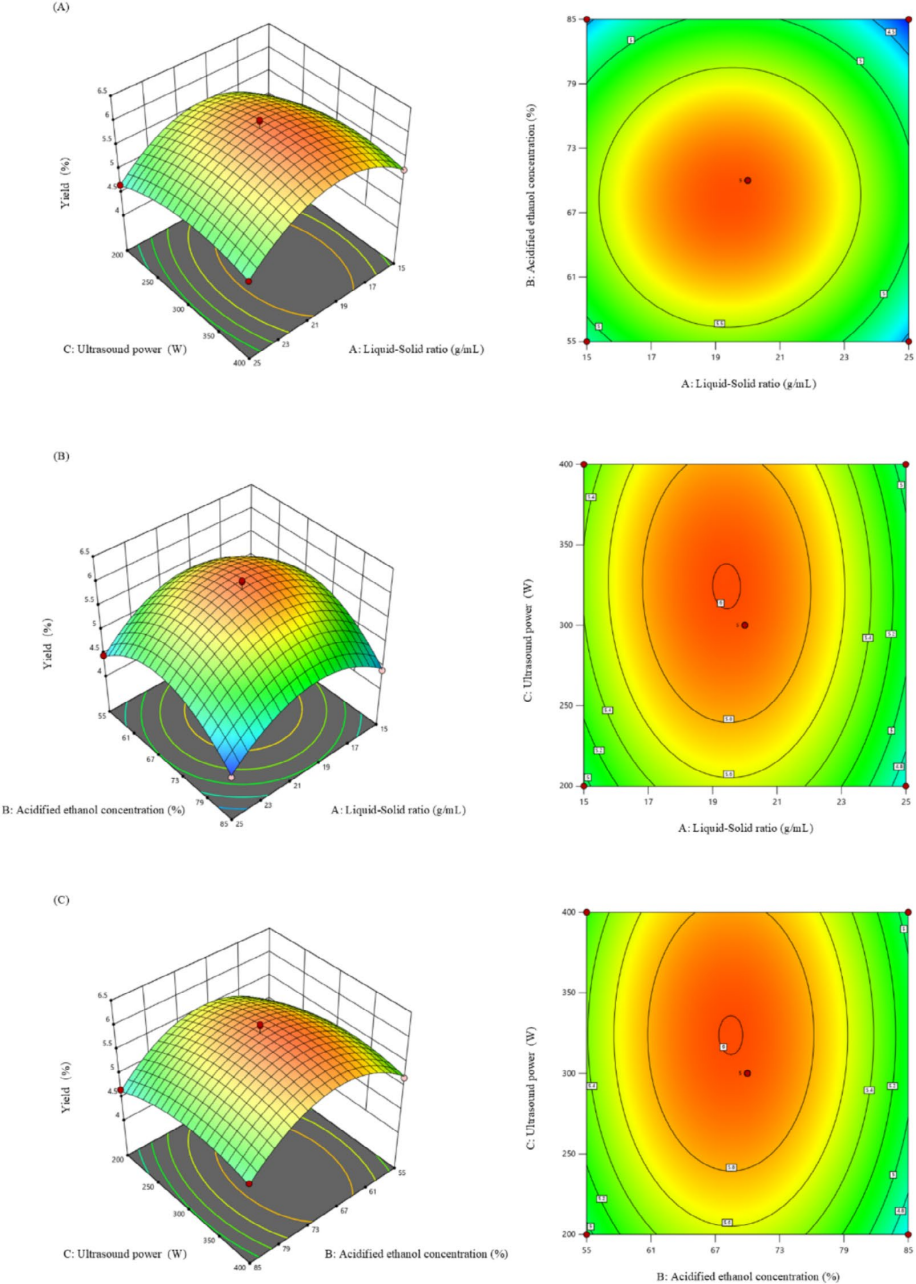
3D map of the response surface and two‐dimensional (2D) contour plot of mulberry anthocyanins.

Response surface and contour plots can visualize the interaction between different factors and the degree of influence (Guo et al. 2024). The following figure shows the response surface analysis of the independent variables with significant interaction effects analyzed by regression variance analysis. Figure 2a–c represents the effect of interaction between different extraction factors on mulberry anthocyanins (Ahmad et al. 2015). The experimental results were basically consistent with the methodological analysis. As shown in Figure 2a, the steepness of the curves of solid–liquid ratio (A) and ultrasonic power (C) was greater for material‐liquid ratio, indicating that the effect of the response surface was higher in the interaction of the two factors. Figure 2b represents the results of the interaction between feed‐liquid ratio and ethanol concentration, from which it can be found that the slope of the curve in the direction of solid–liquid ratio is steeper than that of ethanol concentration, which indicates that solid–liquid ratio has a higher effect on the extraction rate among the two groups of factors. In addition, the contour plots show an elliptical shape, indicating that the interaction between solid–liquid ratio and ethanol concentration is more significant. Figure 2c represents the interaction of ethanol concentration (B) and ultrasonic power (C), which shows that the interaction of ethanol concentration and ultrasonic power is more significant, while the ethanol concentration (B) is steeper than the ultrasonic power (C), which proves that this factor has a higher degree of influence on the extraction rate of anthocyanins from mulberry. The effects of the three on the extraction rate of mulberry anthocyanins were solid–liquid ratio > ethanol concentration > ultrasonic power.

Based on the above model, the optimal conditions for the extraction of mulberry anthocyanins were as follows: material‐liquid ratio 1:20, ethanol concentration 80%, ultrasonic power 340 W. In order to test the reliability of the predicted response values, the optimized conditions were chosen as follows: material‐liquid ratio 1:20, ethanol concentration 80%, ultrasonic power 340 W. Three repetitions of the experiments were carried out, and the obtained content of mulberry anthocyanin was 5.98 mg/g. The results were close to the predicted values, which proved that the optimized results were more reliable. The content of MA was 5.98 mg/g, which was basically close to the predicted value, proving that the optimized results were more reliable (Rocha et al. 2020). Xie et al. (2018) extracted mulberry seeds from four different sources by SEM. The extraction temperature was 20°C, the extraction time was 44.95 min, and the concentration of β‐CD was 45 g L^−1^. The final results showed that the yield of mulberry anthocyanin was 1.2 mg/g. Chen et al. (2023) used the ultrahigh‐pressure extraction method to extract mulberry from mulberry. When the extraction pressure was 429.52 MPa and the solid–liquid ratio was 12.371:1, the final mulberry anthocyanin content was 1.97 mg/g. Guo et al. (2019) used the method of ultrasound‐assisted deep eutectic solvent extraction to obtain mulberry anthocyanin content of 6.05 mg/g under the conditions of extraction time of 30 min, solid–liquid ratio of 22 mL/g, and negative pressure of 0.08 MPa. (Li et al. 2020) (Guo et al. 2019) used the enzyme‐assisted extraction method in mulberry wine residue according to the solid–liquid ratio of 1:20, the extraction time was 58 min, the pH value was 5.9, and the extraction temperature was 45°C. The final mulberry anthocyanin content was 6.04 mg/g. The above results show that the UAME method, as a new extraction method, has a better extraction effect.

### 3.4 In Vitro Antioxidant Activity of Anthocyanins From Mulberry

#### 3.4.1 Ability to Scavenge DPPH·, ABTS·, ·OH Radicals

The activity of DPPH· free radical scavenging in the sample can be used as an important index to evaluate the biological activity of the sample (Xu et al. 2020). After adding mulberry anthocyanins to the DPPH· solution, the solution color can be converted to light yellow because the H atoms provided by the anthocyanins can be paired with the lone pair of electrons in the DPPH·. Hydroxyl radicals are harmful reactive oxygen radicals that react with most of the biomolecules in the body's cells, such as proteins, nucleic acids, and polysaccharides. It has been pointed out that hydroxyl radicals are inextricably linked to DNA strand breaks, and this link is the basis for the promotion and generation of cancer (Miller et al. 1996). Therefore, the current research on the scavenging of hydroxyl radicals is of great significance. At the same time, the free radical scavenging rate of ABTS· was measured to determine its total antioxidant capacity.

**FIGURE 3:**
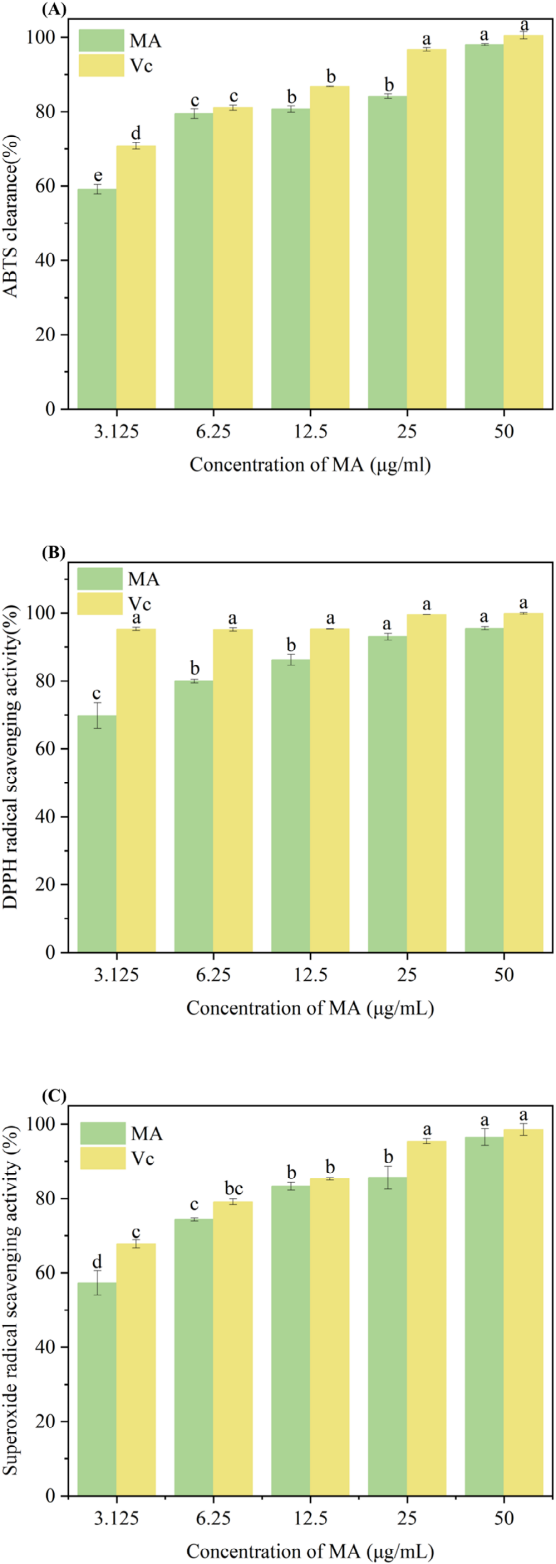
Scavenging ability of MA on DPPH· (A), ABTS· (B), and OH· (C) radicals; different letters indicate significant differences between groups (*p* < 0.05).

As shown in Figure 3, the scavenging of DPPH· radicals by both mulberry anthocyanins and Vc was continuously enhanced with increasing concentration. When the concentration of mulberry anthocyanins reached 50 μg/mL, the scavenging rate of DPPH· free radicals by both it and Vc reached more than 95%. It proved that mulberry anthocyanins had better scavenging ability for DPPH· radicals. For ABTS· radicals, the scavenging rate of both of them increased with the increase of concentration, and the scavenging effect of both mulberry anthocyanins and Vc reached a better scavenging effect at 3 μg/mL, and the scavenging effect of both of them was basically the same, which was up to more than 98% on ABTS·. The scavenging effect of mulberry anthocyanins on ·OH radicals was better, and the scavenging ability was much larger than that of Vc, with a scavenging rate of 97% at 0.1 μg/mL. The above results indicated that mulberry anthocyanins have strong ability against DPPH·, ABTS·, and ·OH radicals. Wang et al. (2024) explored the scavenging rate of mulberry anthocyanins on FRAP and DPPH· free radicals, and found that their scavenging abilities were (96.4% ± 0.76%) and (82.52% ± 2.13%), respectively. At the same dose, its antioxidant effect was higher than that of Vc. Chen et al. (2024) (Wang et al. 2024) found that the extract of mulberry anthocyanins had an effect on DPPH· and ABTS· scavenging, and found that the IC_50_ range of DPPH· scavenging of mulberry anthocyanins extracted by 60% ethanol was 0.056–0.180 mg/mL. Liu et al. (2019) also demonstrated that natural pigments from edible mushroom residues exhibit strong scavenging capacities against free radicals, which supports the conclusion that plant‐derived bioactive substances like mulberry anthocyanins possess significant antioxidant potential. Huang et al. (2024) studied the protective effect of mulberry anthocyanins on H_2_O_2_‐induced vascular endothelial cell (VEC) injury. It was found that mulberry anthocyanins could restore the decrease of vascular cell viability induced by H_2_O_2_, increase superoxide dismutase (SOD), and decrease malondialdehyde (MDA) and xanthine oxidase (XO^−1^) activity.

### 3.5 Analysis of 4 T1 Cell Survival by MA

**FIGURE 4:**
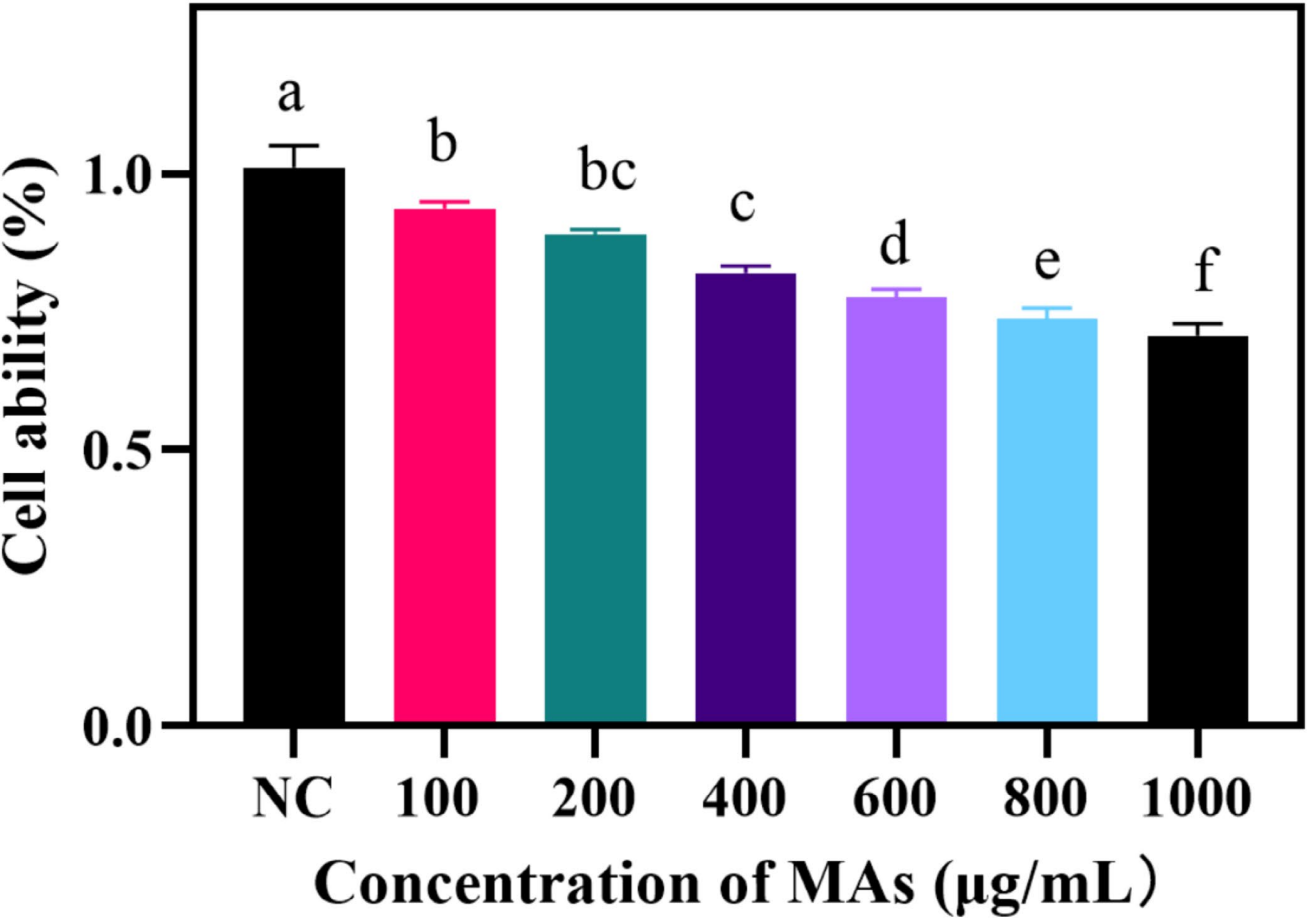
MA on the viability of 4 T1 cells for 24 h. All values are expressed as the mean standard deviation (*n* = 3). Significant differences between different letters (*p* < 0.05).

In order to explore the effect of MA on 4 T1 survival, the CCK8 kit was used in this experiment to explore the effect of different concentrations of MA on 4 T1 cells (Wen et al. 2024). The results are shown in Figure 4, where different concentrations of MA had a dose‐dependent inhibitory effect on 4 T1 cells compared with the control group. At the low concentration interval, the viability of 4 T1 cells was maintained at a high level, indicating that the inhibitory effect of mulberry anthocyanins was weak at this time and could not significantly inhibit the proliferation of 4 T1 cells. This may be because the low concentration of anthocyanins is difficult to trigger significant cytotoxic effects or induce apoptosis. As the concentration increased, the survival rate of 4 T1 cells decreased significantly, showing a strong inhibitory effect. In this concentration range, mulberry anthocyanins may exert antitumor effects by inducing oxidative stress or interfering with the cell cycle to block 4 T1 cell proliferation.

### 3.6 Effects of Different Concentrations of Mulberry Anthocyanin on the Morphology of 4 T1 Cells

**FIGURE 5:**
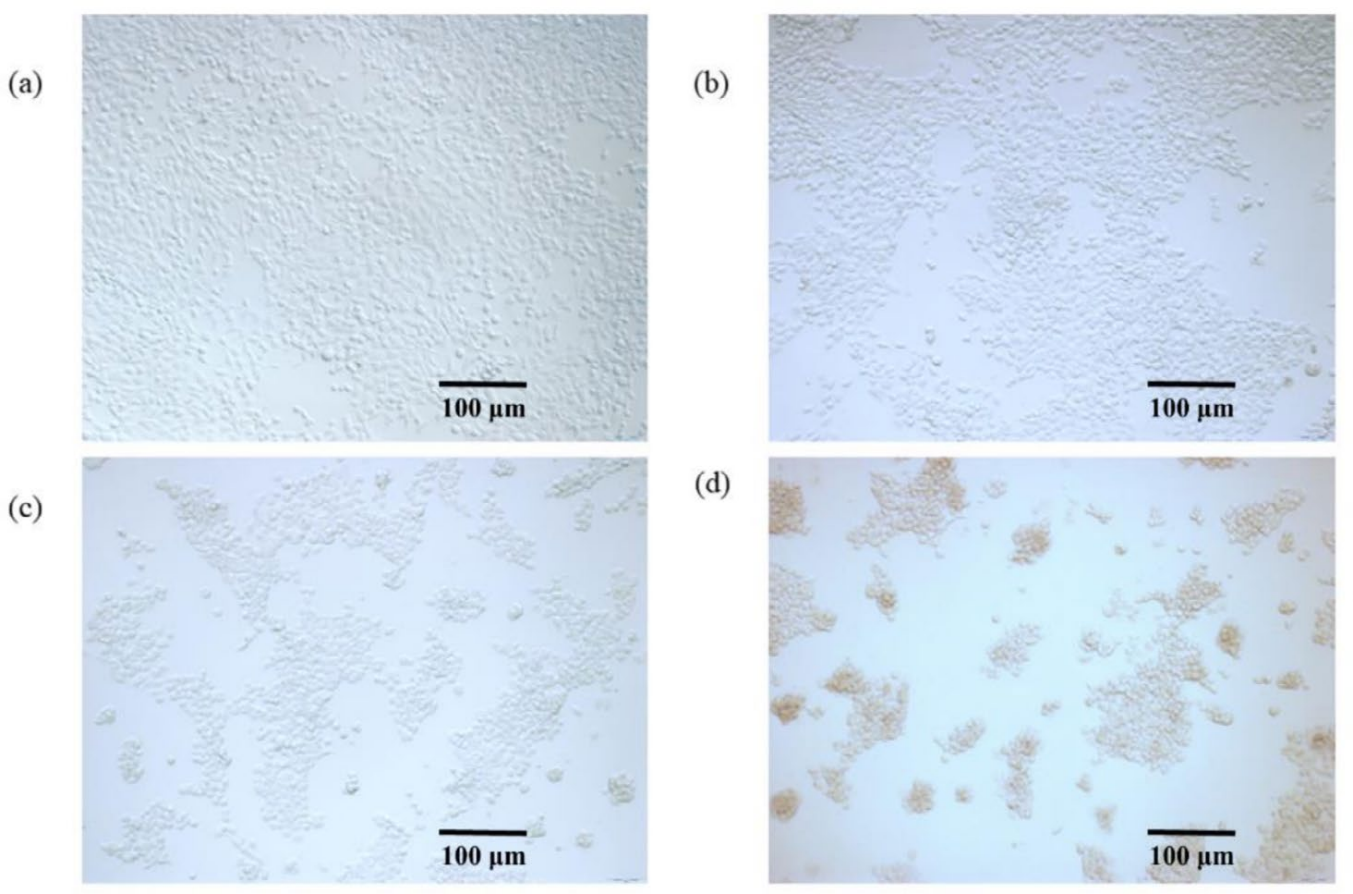
Morphological effects of different mulberry anthocyanin concentrations on 4 T1 cells (a) Normal control group of 4 T1 cells, (b) 250 μg/mL MA on 4 T1 cells, (c) 500 μg/mL MA on 4 T1 cells, (d) 1000 μg/mL MA on 4 T1 cells.

Drugs act on tumor cells to induce apoptosis, and the cells undergo morphological changes such as shrinkage, nuclear chromatin condensation, blistering of the cell membrane, and cellular fragmentation (Xie et al. 2023). As shown in Figure 5, there were different changes in cell density and morphology after the intervention of different concentrations of mulberry anthocyanins compared to the control group (a). In 250 μg/mL (b), the density of the cells after the intervention of mulberry anthocyanin was reduced compared with the control group, and there was no obvious change in the cell morphology; at 500 μg/mL (c), some cells appeared to be agglomerated, and apoptosis appeared after the intervention of the drug, and the morphology of the cells after the intervention of the drug at 1000 μg/mL (d) showed that the cells were obviously agglomerated, irregular in shape, and ruptured cell membranes, and there was an obvious decrease in the cell density compared with the control group. The cell density was significantly decreased compared with the control group. The results demonstrated that MA significantly altered the morphology of 4 T1 cells, manifesting as distinct cellular shrinkage, membrane disintegration, and prominent cell aggregation, further confirming the potent anti‐4 T1 activity of MA.

### 3.7 Effect of MA on 4 T1 Cell Invasion

**FIGURE 6:**
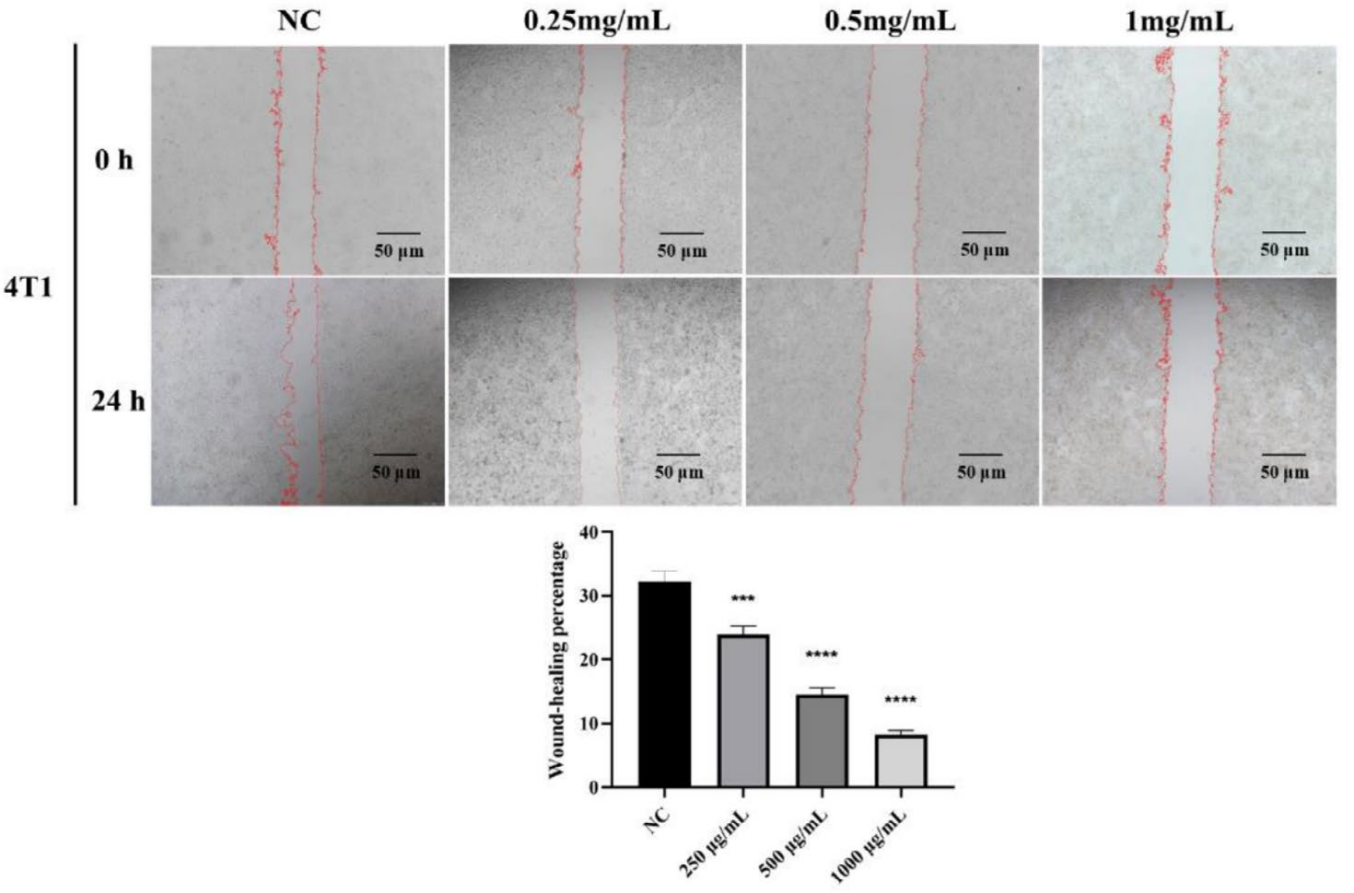
Effect of 250, 500, and 1000 μg/mL of anthocyanins on the migratory ability of 4 T1 cells (All values are expressed as mean (*x* ± *s*) standard deviation (*n* = 3)). (***p < 0.001, ****p < 0.0001).

The effects of different concentrations of MA on the invasion ability of 4 T1 cells are shown in Figure 6. As can be seen from the figure, compared with the blank control group, except for 250 μg/mL of MA, which significantly inhibited the invasion of 4 T1 cells (*p* < 0.001), the rest of the concentrations of MA inhibited the invasion of 4 T1 cells extremely significantly (*p* < 0.0001). It was not difficult to find that the healing rate of 4 T1 cell scratches was gradually decreasing with the increase in the concentration of MA, and the wound healing rate of 4 T1 cells was the lowest when its concentration reached 1000 μg/mL. The results showed that the invasion ability of 4 T1 cells decreased with the increase in drug concentration. Therefore, it was proved that MA had a good function of inhibiting cell invasion and could inhibit the metastasis and invasion of 4 T1 cells.

### 3.8 Detection of Apoptosis by Annexin V‐FITC/PI Double Staining Assay

**FIGURE 7:**
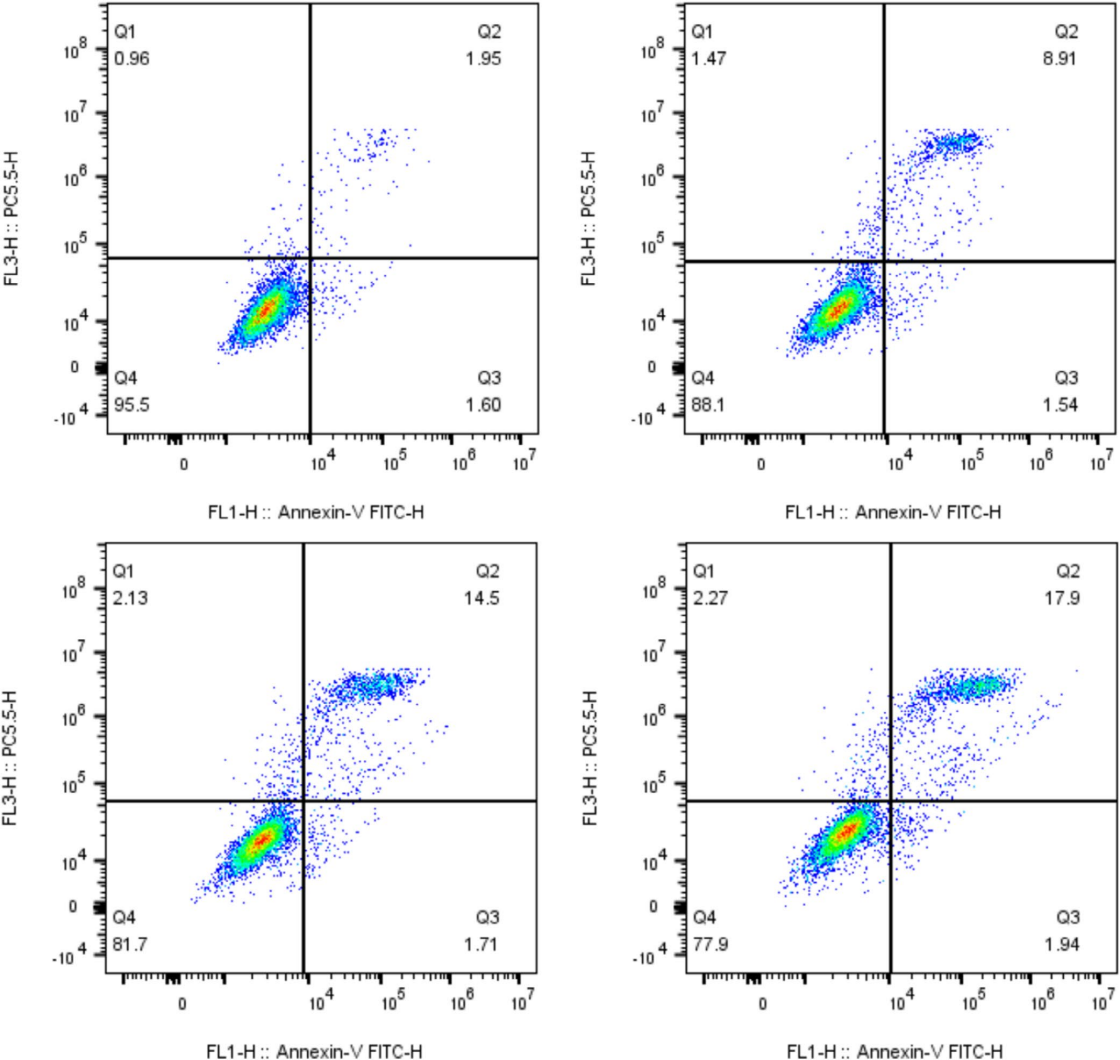
Effect of different concentrations of anthocyanins on apoptosis rate of 4 T1 cells (Effect of 250, 500, and 1000 μg/mL of MA on the apoptosis of 4 T1 cells).

In cell biology, apoptosis is a regulated process of programmed cell death that plays a key role in the maintenance of organismal development within the cells of living organisms, in the maintenance of tissue homeostasis, and in disease. Flow cytometry is used in the analysis of cell cycle and apoptosis (Manohar et al. 2021). In this experiment, flow cytometry was employed to analyze the effects of MA on the 4 T1 cell apoptosis. As shown in Figure 7a–d, represent the effects of MA on the apoptosis level of 4 T1 cells in the blank group, 0.25, 0.5, and 1 mg/mL, respectively. Few cells in the blank group (a) were apoptotic, which might be considered as apoptosis caused by mechanical effects during the operation. A significant increase in the early and middle apoptosis rate of cells was found in the 0.25 mg/mL (b) group. With the increasing concentration of MA, the apoptosis rate of 4 T1 cells also increased gradually, and it can be hypothesized that MA has the effect of promoting apoptosis of 4 T1 cells significantly (Xie et al. 2023).

## 4 Conclusion

In this experiment, ultrasound‐microwave assisted extraction (UMAE) was mainly used to extract anthocyanins from mulberry. It was found that the content of mulberry anthocyanins was as high as 5.98 mg/g at a material‐liquid ratio of 1:20, an ethanol concentration of 80%, and an ultrasonic power of 340 W. This experiment also explored the effects of MA on the activity, cell morphology, apoptosis, and cell migration of the 4 T1 cells, and it was found that, with the elevation of the concentration of MA, the activity of the 4 T1 cells declined, the morphology of the cells was altered, the degree of cell apoptosis rose, and the invasiveness of cells was weakened. This experiment also demonstrated that MA extracted by the UMAE method exhibits antioxidant capacity similar to that of Vc. This indirectly verifies the health–promoting functions of mulberries in the human body. Mulberries can play a role in scavenging free radicals in the human body and delaying aging. This experiment provides a solid theoretical basis for later research on the extraction of mulberry anthocyanins and their biological activities, as well as a certain reference for the medical industry and food industry. Future research will focus on the specific intracellular molecular mechanism of breast cancer in order to achieve a better anticancer effect.

## Author Contributions

**Haibao Tang:** software (equal), writing – original draft (lead), writing – review and editing (equal). **Baogang Zhou:** software (equal), writing – original draft (equal). **Ran Liu:** software (equal), supervision (equal). **Luo Weng:** software (equal), visualization (equal). **Kundian Che:** software (equal), visualization (equal). **Wei Gao:** software (equal). **Zhanjun Chen:** validation (equal). **Jialin Yang:** software (equal). **Haoyuan Luo:** software (equal). **Shangjian Li:** supervision (equal). **Wenzhong Hu:** resources (equal).

## Conflicts of Interest

The authors declare no conflicts of interest.

## Data Availability

Data will be provided availability on request.
